# Supplementary material for: Congenital heart disease detection by pediatric electrocardiogram based deep learning integrated with human concepts
Source: Nat Commun. 2024 Feb 1;15:976. doi: 10.1038/s41467-024-44930-y (PMC10834950; doi:10.1038/s41467-024-44930-y)
Supplement: Supplementary file 1 — Supplementary Information [file 41467_2024_44930_MOESM1_ESM.pdf]

Supplementary Materials

Detailed Reasons of Data Collection and Sampling

This study collected and used pediatric electrocardiogram (ECG) data from different individuals, and parts of the cases were discarded due the following reasons.

- We excluded the cases with corrupted ECG signals or heavy noises. Pediatric ECG signals are typically more noisy than adult ECG data, as pediatric patients often have poor control over their behaviors during ECG examinations and thus more noisy signals may be included (e.g., myoelectric signals). The ECG cases are excluded if over 20% signal values exceed 5 mV or if over 20% signal values are 0's.
- Since we used the diagnostic results as ground truth (classification labels) to train and validate our models, we excluded cases with missing diagnostic results.
- We excluded the ECG data recorded after interventions, since our aim was to study detection of CHDs for interventions.
- In order to avoid introducing biases of undesired oversampling on some individuals, we excluded the ECG data from the individuals whose other ECG data had been used in the study before partitioning the training, testing, and validation sets. Regarding the external test set from Center-B, we also excluded ECG data from individuals present in datasets from Center-A. Moreover, the selection process for individuals in the Center-B external test set involved meticulous information filtering based on the the enterprise master patient index (EMPI) of the hospital. The individuals included in the Center-C external test set underwent a similar information filtering process, employing criteria such as age, date of birth, sex, and name. This approach effectively guarantees the absence of overlapping individuals. The ECG data and corresponding diagnostic results from the last examinations were utilized for these selected individuals.

Sample Characteristics

In this study, a total of 65,869 cases were employed for model training and validation. Additionally, 12,000 cases were utilized to form the internal test set, and two external test sets comprise 7,137 and 8,121 cases, respectively. We presented the demographic and clinical characteristics of these cohorts in Table S1, which provides a summary of distributions in term of age, sex, ECG features, and CHD subtypes. Basic demographic features such as age and sex showed good consistency across four different cohorts. Significant difference of CHD diagnostic types from two externally independent test sets gives us the possibility to more broadly validate the CHDdECG model.

Formulas for Computing the Human Knowledge Features

A total of 114 clinically useful human-concept features were computed to present human knowledge. The detailed formulas for these 114 features were listed in Table S2.

Model Performance Comparison

We compared the model performances of our proposed CHDdECG with other models, including 1D convolutional neural network (CNN) following the setting of Hannun's work[1], long-short term memory (LSTM)[2] with default hyperparameters, k-nearest neighboring classifier ($k = 2$), and carefully tuned XGboost model[3] and random forest model (RF)[4]. Since these models are designed to use single feature type, the 1D CNN and LSTM were fed with ECG signals after the pre-processing. While the k-NN, XGboost and random forest models are conducted on human knowledge features in the tabular data format. The performances measured by ROC-AUC and PR-AUC are reported in Table S3 and Table S4. All of these results indicate that our proposed CHDdECG outperforms other AI approaches by considerable margins.

Comparative Impact Study of Feature Types

The proposed CHDdECG utilizes three feature types for comprehensive prediction. In this study, we conducted comparative experiments on the test set to examine the contributions of various feature types in deploying CHDdECG, using all or parts of the feature types. The results are illustrated in Figure S1, which demonstrate that the model using all three feature types performs best in general. Specifically, the recall rate is critical in CHD detection, and we observed that CHDdECG using all three feature types achieves considerably better recall rates than other settings. Additionally, it is noteworthy that the model performances with only wave features, clinical features, or ECG signals are all not too bad, suggesting that all three feature

types are informative. It is also observed that the model using only ECG signals outperforms the model using only clinical features, which further outperforms the model using only wavelet features.

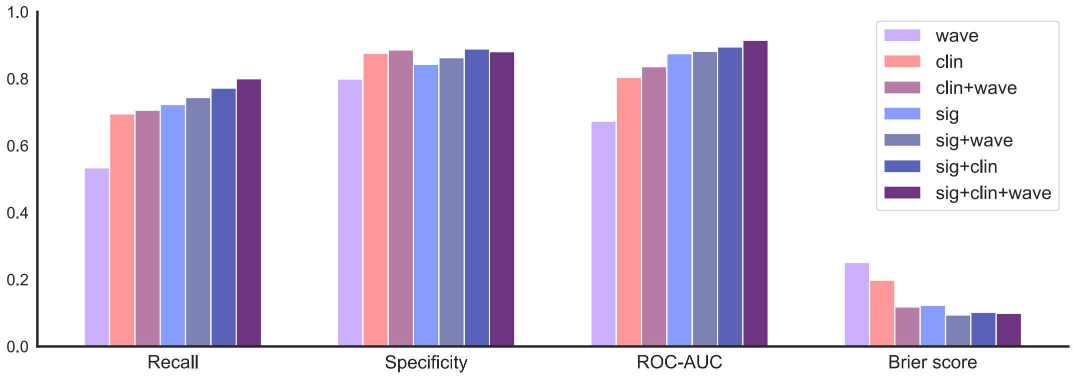

Figure S1. An illustration of ablation study results, measured by recall (↑), specificity (↑), ROC-AUC (↑), brier score (↓). The legend indicates the used feature types in comparison, where "sig", "clin", and "wave" denote "ECG signals", "clinical features", and "wavelet features", respectively.

Individual Stratification Characteristics and Stratified Performance Verification

The CHDdECG's performance was checked further in terms of stratification by patient age, sex, and heart rates (see Table S5). On the internal and two external test sets, the CHDdECG model consistently excels across sex, age, and heart rate stratifications, demonstrating a uniform and robust level of effectiveness in diverse characteristics.

Net Reclassification Index (NRI) Analysis

As shown in Figure S2(a), the NRI scores of the CHDdECG model and cardiologists assisted by CHDdECG identified activated segments, compared with the performance of cardiologists without any assistance as the benchmark, indicate that CHDdECG demonstrates superior CHD detection capabilities in comparison to cardiologists and demonstrated that CHDdECG has the potential to enhance the diagnostic performance of cardiologists. The NRI(+) and NRI(-) scores are depicted in Figure S2 (b) and (c), respectively. NRI(+) scores reveal a significant contribution in enhancing the NRI values. Combined with the marginal NRI(-) scores, NRI analysis suggests that the high performance of CHDdECG is predominantly attributed to its proficiency in identifying CHD cases (increased sensitivity).

References

1. Hannun, A. Y. et al. Cardiologist-level arrhythmia detection and classification in ambulatory electrocardiograms using a deep neural network. Nat. Medicine (2019).
2. Hochreiter, S. & Schmidhuber, J. Long short-term memory. Neural Comput. (1997).
3. Chen, T. & Guestrin, C. Xgboost: A scalable tree boosting system. In ACM SigKDD International Conference on Knowledge Discovery and Data Mining (2016).
4. Breiman, L. Random forests. Mach. Learn. (2001).

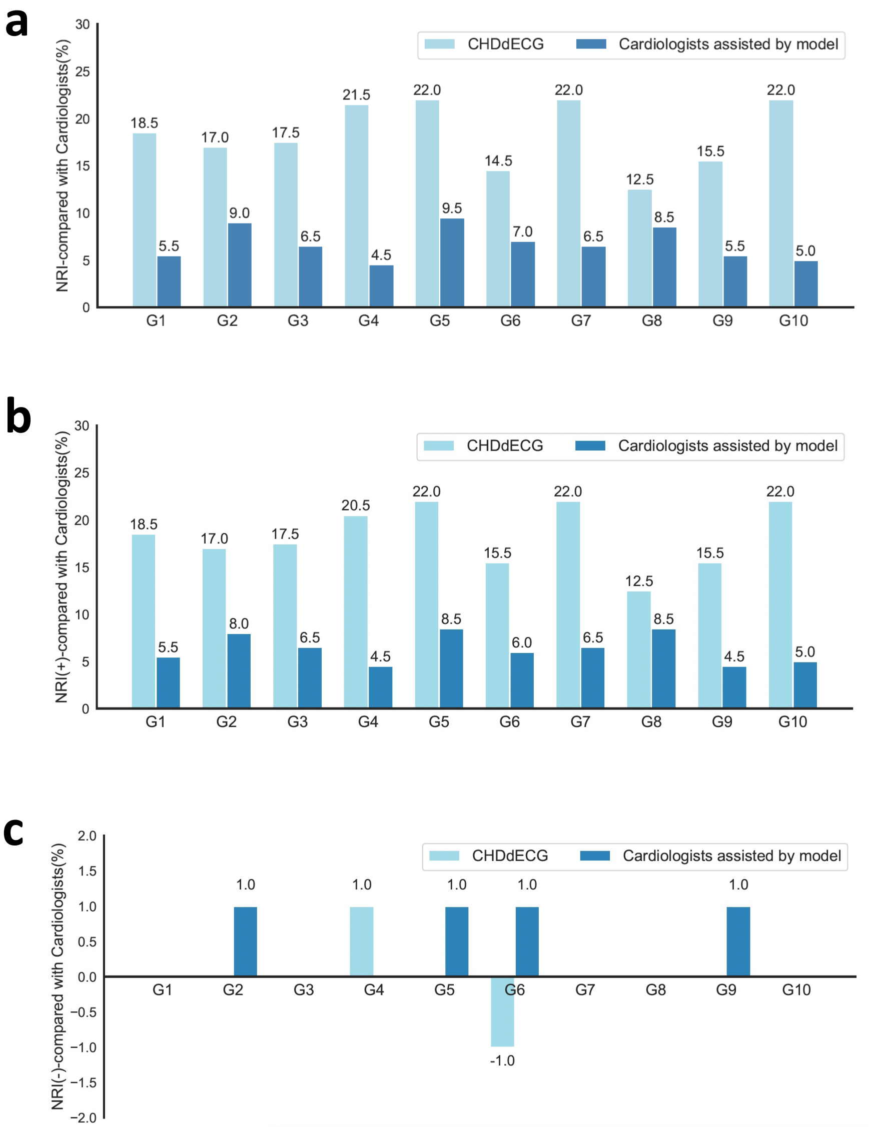

Figure S2. (a) illustrated NRI of CHDdECG and cardiologists assisted by CHDdECG, compared with cardiologists without any assistance as benchmark, across 10 randomly sampled test data groups from the Center-A test set. (b) and (c) illustrated the corresponding NRI(+) and NRI(-).

Characteristics		Training & validation set from Center-A	Test set from Center-A	External test set from Center-B	External test set from Center-C
Age (years):		2.12 ± 1.50	2.13±1.23	1.55±1.04	1.95±1.17
Sex (sample count (proportion)):					
	Male	41996 (63.8%)	7758 (64.65%)	3679 (51.55%)	4398 (54.16%)
	Female	23873 (36.2%)	4242 (35.35%)	3458 (48.45%)	3723 (45.84%)
ECG Features (mean ± std):					
	P wave apex values of lead I (uV)	47.2±30.6	47.4±30.8	40.4±24.8	63.6±42.2
	P wave apex values of lead II (uV)	47.2±28.5	47.2±29.2	42.1±24.1	66±46.3
	P wave apex values of lead III (uV)	32.7±24.9	32.3±25	28.4±20.3	43.9±36.2
	P wave apex values of lead aVR (uV)	62.9±25.3	63±25.7	59±21.6	84.7±45
	P wave apex values of lead aVL (uV)	35±27.3	34.8±27.5	29.8±21.4	46.4±35.4
	P wave apex values of lead aVF (uV)	35.8±25.3	35.2±24.9	31.9±21.4	48.4±37.8
	P wave apex values of lead V1 (uV)	69.7±30.8	70.2±30.9	62.9±26.1	85±42.5
	P wave apex values of lead V3 (uV)	53.7±48.8	53.4±48.7	23.9±45.4	63.1±60.4
	P wave apex values of lead V5 (uV)	19.7±55.4	19.8±55.5	5.7±42.6	25.1±63.7
	Q wave apex values of lead I (uV)	-67.8±35.7	-68±36	-58.7±27.1	-85.3±51.7
	Q wave apex values of lead II (uV)	-111.7±48.8	-112.4±49.4	-111.1±45.2	-138.3±70.9
	Q wave apex values of lead III (uV)	-117±74.8	-118.5±75.4	-112.3±68.4	-153.4±116.3
	Q wave apex values of lead aVR (uV)	-223.1±116.7	-221.2±115.9	-204.2±108.9	-433.3±352.5
	Q wave apex values of lead aVL (uV)	-46.1±37.6	-45±36.2	-35.3±24.9	-57.6±52.4
	Q wave apex values of lead aVF (uV)	-102.4±56.7	-103.2±57.6	-102.4±53.8	-125.9±77.4
	Q wave apex values of lead V1 (uV)	-48.8±39.2	-48.2±39.5	-39.7±30.1	-63.8±55.4
	Q wave apex values of lead V3 (uV)	-100.8±56.5	-101.3±57.2	-111.5±50.8	-123.2±79.4
	Q wave apex values of lead V5 (uV)	-148.9±71	-148.7±70.3	-128±53.4	-180.7±97.1
	R wave apex values of lead I (uV)	267.4±109.4	269.4±111.4	222.1±90.8	448.7±309.9
	R wave apex values of lead II (uV)	399.3±148.3	400±149.7	376.2±142.6	628.4±388.1
	R wave apex values of lead III (uV)	323.3±160.7	325.5±161.6	301±149.2	454.2±289.1
	R wave apex values of lead aVR (uV)	180.5±75.5	181±76.9	150.6±53.6	294.9±194.6
	R wave apex values of lead aVL (uV)	174.7±89.4	175.9±90.1	135.4±62.5	272.6±195.5
	R wave apex values of lead aVF (uV)	335.2±148	336.1±149	318.1±140.8	487.5±298
	R wave apex values of lead V1 (uV)	435.9±180.3	438.1±181.4	430±166.6	610.4±374.4
	R wave apex values of lead V3 (uV)	770.2±230.5	771.1±228.4	789±222.5	1261.3±803.1
	R wave apex values of lead V5 (uV)	653.5±229.9	650.1±226.2	533±186	1048.4±672.5
	S wave apex values of lead I (uV)	-171.4±90.9	-171.2±90.6	-154.7±76.8	-285.6±225.4
	S wave apex values of lead II (uV)	-150.7±62.8	-149.9±62.4	-138.8±54.6	-265.6±195.9
	S wave apex values of lead III (uV)	-77.7±39.8	-77.5±40.6	-69±31.4	-128.8±96.2
	S wave apex values of lead aVR (uV)	-72.8±91.7	-74.6±92	-63.8±81.8	-65.2±85.3
	S wave apex values of lead aVL (uV)	-166.1±111	-167.6±112	-159.6±102.4	-241.8±200.4
	S wave apex values of lead aVF (uV)	-102.5±45.2	-102±45	-93.7±38.1	-174.3±128
	S wave apex values of lead V1 (uV)	-337.8±187.7	-340.1±189.2	-325.5±169.3	-443.7±289.5
	S wave apex values of lead V3 (uV)	-573.7±255	-571.1±252.2	-505.5±214.5	-931.6±656
	S wave apex values of lead V5 (uV)	-281.3±126.2	-280.4±125.2	-224.5±89.2	-489.9±374
	T wave apex values of lead I (uV)	78.3±42.4	77.9±42.5	66.8±38.2	107.2±62
	T wave apex values of lead II (uV)	90±43.4	90.2±43.8	81.7±40	120.1±67.2
	T wave apex values of lead III (uV)	29±29.5	29.1±30.2	20.7±24.1	46.7±48.3
	T wave apex values of lead aVR (uV)	45±28.5	45±28.6	38.7±24.8	60.3±36
	T wave apex values of lead aVL (uV)	44.5±27.4	44.3±27.1	35±19.1	67.9±48.5
	T wave apex values of lead aVF (uV)	53.4±33.7	53.4±33.9	46.4±29.5	75.3±52.4
	T wave apex values of lead V1 (uV)	55±43.9	54.7±44.8	41±42.2	78±66
	T wave apex values of lead V3 (uV)	84.8±54.8	84.1±55.4	81.6±53.4	125.6±93.3
	T wave apex values of lead V5 (uV)	124.4±74.2	124.7±73.9	107.6±59.1	163.5±96.7
	P-P interval duration (ms)	488±99.8	487.2±100.2	447.6±82.6	505.6±113.8
	QRS complex duration (ms)	168±40.2	169±40.8	163.8±38	148.4±45
	R-R interval duration (ms)	488.4±100.2	486.8±99.8	447±81.8	505.6±113.8
	S-T interval duration (ms)	127±43	126.4±43.2	135.6±40.4	151.2±55.6
	ST segment duration (ms)	44.8±31.4	44.2±31.4	20.8±15.8	46.2±28.6
	Q-T interval duration (ms)	297.8±36.2	298.2±37.4	303±32.8	302.4±38.6
	P-R interval duration (ms)	77.6±29.2	78.4±29.6	89.2±29.4	88.6±35.6
	PR segment duration (ms)	25±16.6	25±16.6	15.4±13	25±16.6
	T-T interval duration (ms)	488±99.4	487±99.4	446.8±81.6	505.2±113.4
	duration of P wave (ms)	58.4±27.8	58.8±27.8	87.8±28.6	67.6±29.8
	duration of T wave (ms)	87.6±18.6	87.6±18.6	128.6±25.2	107.8±36.6
CHD subtypes (sample count (proportion)):					
	Atrial septal defect	1311 (12%)	248 (12.2%)	85 (28.3%)	1315 (52.2%)
	Patent ductus arteriosus	710(6.5%)	136 (6.7%)	36 (12%)	199 (7.9%)
	Anomalous origin of a coronary artery	68 (0.6%)	18 (0.9%)	/	15 (0.6%)
	Ventricular septal defect	3975(36.4%)	745 (36.6%)	100 (33.3%)	589 (23.4%)
	Coarctation of the aorta	246 (2.3%)	38 (1.9%)	/	24 (1%)
	Total anomalous pulmonary venous connection	141 (1.3%)	17 (0.8%)	/	9 (0.4%)
	dextro-Transposition of the great arteries	270 (2.5%)	43 (2.1%)	2 (0.7%)	11 (0.4%)
	Pulmonary atresia	248 (2.3%)	51 (2.5%)	3 (1.0%)	57 (2.3%)
	Single ventricle	106 (1%)	19 (0.9%)	/	15 (0.6%)
	Tetralogy of fallot	370(3.4%)	68(3.3%)	/	92 (3.6%)
	Atrioventricular septal defect	169 (1.5%)	40 (2.0%)	/	18 (0.7%)
	Double-outlet right ventricle	101 (0.9%)	16 (0.8%)	2 (0.7%)	9 (0.4%)
	Congenital Mitral Valve Insufficiency	34 (0.3%)	10 (0.5%)	2 (0.7%)	8 (0.3%)
	Congenital Aortic Arch Disruption	33 (0.3%)	11 (0.5%)	/	6 (0.2%)
	Tricuspid Atresia	26 (0.2%)	7 (0.3%)	/	/
	Common Arterial Trunk	25 (0.2%)	5 (0.2%)	4 (1.3%)	/
	Congenital Aortic Valve Stenosis	13 (0.1%)	3 (0.1%)	/	5 (0.2%)
	Ebstein's anomaly	11 (0.1%)	3 (0.1%)	/	5 (0.2%)
	Congenital Tricuspid Valve Insufficiency	12 (0.1%)	2 (0.1%)	/	/
	double chamber right ventricle	10 (0.1%)	2 (0.1%)	/	6 (0.2%)
	Coronary Artery to Right Ventricle Fistula	8 (0.1%)	2 (0.1%)	2 (0.7%)	/
	Double Aortic Arch	7 (0.1%)	2 (0.1%)	/	9 (0.4%)
	Congenital Coronary Artery Anomaly	7 (0.1%)	2 (0.1%)	/	/
	Congenital Tricuspid Valve Atresia	6 (0.1%)	2 (0.1%)	/	/
	Ventricular Noncompaction Cardiomyopathy	6 (0.1%)	2 (0.1%)	/	/
	Fallot's Pentalogy	6 (0.1%)	2 (0.1%)	/	5 (0.2%)
	Coronary Artery to Right Atrium Fistula	6 (0.1%)	2 (0.1%)	/	8 (0.3%)
	Congenital Coronary Artery to Pulmonary Artery Fistula	6 (0.1%)	2 (0.1%)	2 (0.7%)	9 (0.4%)
	unknown sub-types	2996 (27.4%)	540 (26.5%)	62 (20.7%)	107 (4.2%)

Table S1. Demographic and clinical characteristics of cohorts. Values are represented in mean ± standard deviation (minimum–maximum) or count (proportion, %).

Feature Definitions	Rule Formulas
(1) mean value of P wave apex values, $\bar{V}(\mathrm{P})$	$\bar{V}(\mathrm{P}) = \Sigma_{i=1}^{N} V(\mathrm{P}_i) / N$
(2) mean value of Q wave apex values, $\bar{V}(\mathrm{Q})$	$\bar{V}(\mathrm{Q}) = \Sigma_{i=1}^{N} V(\mathrm{Q}_i) / N$
(3) mean value of R wave apex values, $\bar{V}(\mathrm{R})$	$\bar{V}(\mathrm{R}) = \Sigma_{i=1}^{N} V(\mathrm{R}_i) / N$
(4) mean value of S wave apex values, $\bar{V}(\mathrm{S})$	$\bar{V}(\mathrm{S}) = \Sigma_{i=1}^{N} V(\mathrm{S}_i) / N$
(5) mean value of T wave apex values, $\bar{V}(\mathrm{T})$	$\bar{V}(\mathrm{T}) = \Sigma_{i=1}^{N} V(\mathrm{T}_i) / N$
(6) ratio of $\bar{V}(\mathrm{P})$ to $\bar{V}(\mathrm{R})$	$r(\mathrm{P}\|\mathrm{R}) = \bar{V}(\mathrm{P}) / \bar{V}(\mathrm{R})$
(7) ratio of $\bar{V}(\mathrm{R})$ to $\bar{V}(\mathrm{Q})$	$r(\mathrm{R}\|\mathrm{Q}) = \bar{V}(\mathrm{R}) / \bar{V}(\mathrm{Q})$
(8) ratio of $\bar{V}(\mathrm{R})$ to $\bar{V}(\mathrm{S})$	$r(\mathrm{R}\|\mathrm{S}) = \bar{V}(\mathrm{R}) / \bar{V}(\mathrm{S})$
(9) ratio of $\bar{V}(\mathrm{T})$ to $\bar{V}(\mathrm{P})$	$r(\mathrm{T}\|\mathrm{P}) = \bar{V}(\mathrm{T}) / \bar{V}(\mathrm{P})$
(10) ratio of $\bar{V}(\mathrm{T})$ to $\bar{V}(\mathrm{R})$	$r(\mathrm{T}\|\mathrm{R}) = \bar{V}(\mathrm{T}) / \bar{V}(\mathrm{R})$
(11) average duration of P wave, $\bar{t}(\mathrm{P})$	$\bar{t}(\mathrm{P}) = \Sigma_{i=1}^{N} (T(\mathrm{P}_i^{(e)}) - T(\mathrm{P}_i^{(o)})) / N$
(12) average duration of T wave, $\bar{t}(\mathrm{T})$	$\bar{t}(\mathrm{T}) = \Sigma_{i=1}^{N} (T(\mathrm{T}_i^{(e)}) - T(\mathrm{T}_i^{(o)})) / N$
(13) average QRS complex duration, $\bar{t}(\mathrm{QRS})$	$\bar{t}(\mathrm{QRS}) = \Sigma_{i=1}^{N} (T(\mathrm{S}_i^{(e)}) - T(\mathrm{Q}_i^{(o)})) / N$
(14) average S-T interval duration, $\bar{t}(\text{S-T})$	$\bar{t}(\text{S-T}) = \Sigma_{i=1}^{N} (T(\mathrm{T}_i^{(e)}) - T(\mathrm{S}_i^{(e)})) / N$
(15) average ST segment duration, $\bar{t}(\mathrm{ST})$	$\bar{t}(\mathrm{ST}) = \Sigma_{i=1}^{N} (T(\mathrm{T}_i^{(o)}) - T(\mathrm{S}_i^{(e)})) / N$
(16) average Q-T interval duration, $\bar{t}(\text{Q-T})$	$\bar{t}(\text{Q-T}) = \Sigma_{i=1}^{N} (T(\mathrm{T}_i^{(e)}) - T(\mathrm{Q}_i^{(o)})) / N$
(17) average P-R interval duration, $\bar{t}(\text{P-R})$	$\bar{t}(\text{P-R}) = \Sigma_{i=1}^{N} (T(\mathrm{Q}_i^{(o)}) - T(\mathrm{P}_i^{(o)})) / N$
(18) average duration of PR segment, $\bar{t}(\mathrm{PR})$	$\bar{t}(\mathrm{PR}) = \Sigma_{i=1}^{N} (T(\mathrm{Q}_i^{(o)}) - T(\mathrm{P}_i^{(e)})) / N$
(19) average P-P interval duration, $\bar{t}(\text{P-P})$	$\bar{t}(\text{P-P}) = \Sigma_{i=2}^{N} (T(\mathrm{P}_i) - T(\mathrm{P}_{i-1})) / (N-1)$
(20) average R-R interval duration, $\bar{t}(\text{R-R})$	$\bar{t}(\text{R-R}) = \Sigma_{i=2}^{N} (T(\mathrm{R}_i) - T(\mathrm{R}_{i-1})) / (N-1)$
(21) average duration of T-T interval, $\bar{t}(\text{T-T})$	$\bar{t}(\text{T-T}) = \Sigma_{i=2}^{N} (T(\mathrm{T}_i) - T(\mathrm{T}_{i-1})) / (N-1)$
(22) ratio of $\bar{t}(\mathrm{P})$ to $\bar{t}(\text{P-R})$	$r(\mathrm{P}\|\text{P-R}) = \bar{t}(\mathrm{P}) / \bar{t}(\text{P-R})$
(23) ratio of $\bar{t}(\mathrm{P})$ to $\bar{t}(\mathrm{PR})$	$r(\mathrm{P}\|\mathrm{PR}) = \bar{t}(\mathrm{P}) / \bar{t}(\mathrm{PR})$
(24) ratio of $\bar{t}(\mathrm{T})$ to $\bar{t}(\text{S-T})$	$r(\mathrm{T}\|\text{S-T}) = \bar{t}(\mathrm{T}) / \bar{t}(\text{S-T})$
(25) ratio of $\bar{t}(\mathrm{T})$ to $\bar{t}(\mathrm{ST})$	$r(\mathrm{T}\|\mathrm{ST}) = \bar{t}(\mathrm{T}) / \bar{t}(\mathrm{ST})$
(26) ratio of $\bar{t}(\mathrm{T})$ to $\bar{t}(\text{Q-T})$	$r(\mathrm{T}\|\text{Q-T}) = \bar{t}(\mathrm{T}) / \bar{t}(\text{Q-T})$
(27) ratio of $\bar{t}(\mathrm{QRS})$ to $\bar{t}(\text{S-T})$	$r(\mathrm{QRS}\|\text{S-T}) = \bar{t}(\mathrm{QRS}) / \bar{t}(\text{S-T})$
(28) ratio of $\bar{t}(\mathrm{QRS})$ to $\bar{t}(\text{Q-T})$	$r(\mathrm{QRS}\|\text{Q-T}) = \bar{t}(\mathrm{QRS}) / \bar{t}(\text{Q-T})$
(29) ratio of $\bar{t}(\text{S-T})$ to $\bar{t}(\text{Q-T})$	$r(\text{S-T}\|\text{Q-T}) = \bar{t}(\text{S-T}) / \bar{t}(\text{Q-T})$
(30) ratio of $\bar{t}(\mathrm{ST})$ to $\bar{t}(\text{S-T})$	$r(\mathrm{ST}\|\text{S-T}) = \bar{t}(\mathrm{ST}) / \bar{t}(\text{S-T})$
(31) ratio of $\bar{t}(\text{P-R})$ to $\bar{t}(\mathrm{QRS})$	$r(\text{P-R}\|\mathrm{QRS}) = \bar{t}(\text{P-R}) / \bar{t}(\mathrm{QRS})$
(32) ratio of $\bar{t}(\text{P-P})$ to $\bar{t}(\text{R-R})$	$r(\text{P-P}\|\text{R-R}) = \bar{t}(\text{P-P}) / \bar{t}(\text{R-R})$
(33) ratio of $\bar{t}(\text{P-P})$ to $\bar{t}(\text{T-T})$	$r(\text{P-P}\|\text{T-T}) = \bar{t}(\text{P-P}) / \bar{t}(\text{T-T})$
(34) ratio of $\bar{t}(\text{R-R})$ to $\bar{t}(\text{T-T})$	$r(\text{R-R}\|\text{T-T}) = \bar{t}(\text{R-R}) / \bar{t}(\text{T-T})$

Table S2. Definitions of the hand-crafted features and their corresponding formulas for computing these features representing expert knowledge for ECG analysis. The features indexed from (1) to (10) were defined on the 9 individual lead signals, and thus 90 ($= 10 \times 9$) scalar features were obtained. The remaining 24 features (from (11) to (34)) were associated with the recording time (i.e., position in the temporal dimension) when the key points of segments occurred. The functions $T(\mathrm{X}^{(o)})$ and $T(\mathrm{X}^{(e)})$ ($\mathrm{X} \in \{\mathrm{P}, \mathrm{Q}, \mathrm{R}, \mathrm{S}, \mathrm{T}\}$) respectively returned the onset time point and end time point of the segment X, and $T(\mathrm{X})$ without a superscript returned the time when the wave apex occurred. The function $V(\mathrm{X})$ returned the voltage value of the segment X. The subscript i of a segment X indicated at which cardiac cycle the segment X located, and N indicated the amount of complete cardiac cycles that the input ECG data contained.

Internal Test Set from Center-A

CHD Subtypes	CHDdECG	CNN	LSTM	k-NN	RF	Xgboost
Atrial septal defect	0.835	0.793	0.815	0.721	0.804	0.819
Patent ductus arteriosus	0.856	0.799	0.835	0.756	0.789	0.803
Anomalous origin of a coronary artery	0.894	0.886	0.852	0.796	0.815	0.901
Ventricular septal defect	0.920	0.912	0.905	0.806	0.855	0.899
Coarctation of the aorta	0.935	0.915	0.930	0.816	0.861	0.923
Total anomalous pulmonary venous connection	0.944	0.920	0.925	0.831	0.886	0.930
dextro-Transposition of the great arteries	0.929	0.891	0.912	0.795	0.849	0.899
Pulmonary atresia	0.985	0.952	0.961	0.864	0.912	0.967
Single ventricle	0.985	0.960	0.966	0.897	0.932	0.990
Tetralogy of fallot	0.987	0.957	0.972	0.883	0.942	0.989
Atrioventricular septal defect	0.991	0.984	0.979	0.868	0.937	0.985
Double-outlet right ventricle	0.992	0.981	0.970	0.872	0.955	0.982
CHD (subtypes not distinguished)	0.915	0.875	0.882	0.777	0.851	0.906

External Test Set from Center-B

CHD Subtypes	CHDdECG	CNN	LSTM	k-NN	RF	Xgboost
Atrial septal defect	0.926	0.887	0.897	0.801	0.873	0.887
Patent ductus arteriosus	0.889	0.862	0.881	0.738	0.823	0.891
Ventricular septal defect	0.918	0.903	0.898	0.797	0.835	0.912
CHD (subtypes not distinguished)	0.917	0.868	0.873	0.754	0.861	0.889

External Test Set from Center-C

CHD Subtypes	CHDdECG	CNN	LSTM	k-NN	RF	Xgboost
Atrial septal defect	0.916	0.903	0.875	0.821	0.889	0.879
Patent ductus arteriosus	0.904	0.786	0.815	0.706	0.853	0.853
Anomalous origin of a coronary artery	0.876	0.843	0.791	0.715	0.801	0.912
Ventricular septal defect	0.913	0.917	0.883	0.821	0.843	0.928
Coarctation of the aorta	0.859	0.801	0.856	0.791	0.782	0.885
Pulmonary atresia	0.906	0.863	0.891	0.815	0.863	0.983
Single ventricle	0.929	0.891	0.913	0.833	0.875	0.935
Tetralogy of fallot	0.939	0.875	0.905	0.874	0.852	0.912
Atrioventricular septal defect	0.909	0.892	0.876	0.881	0.790	0.871
CHD (subtypes not distinguished)	0.907	0.879	0.886	0.781	0.833	0.891

Table S3. Classification performances measured by "ROC-AUC" on internal and external test sets.

Internal Test Set from Center-A

CHD Subtypes	CHDdECG	CNN	LSTM	*k*-NN	RF	Xgboost
Atrial septal defect	0.130	0.088	0.128	0.097	0.132	0.073
Patent ductus arteriosus	0.063	0.028	0.066	0.032	0.084	0.067
Anomalous origin of a coronary artery	0.027	0.020	0.025	0.027	0.020	0.011
Ventricular septal defect	0.540	0.362	0.346	0.141	0.291	0.395
Coarctation of the aorta	0.080	0.053	0.062	0.041	0.074	0.047
Total anomalous pulmonary venous connection	0.054	0.035	0.063	0.056	0.050	0.046
dextro-Transposition of the great arteries	0.096	0.038	0.071	0.065	0.043	0.023
Pulmonary atresia	0.233	0.136	0.148	0.113	0.157	0.133
Single ventricle	0.082	0.052	0.047	0.050	0.058	0.083
Tetralogy of fallot	0.361	0.223	0.241	0.014	0.320	0.242
Atrioventricular septal defect	0.241	0.152	0.175	0.096	0.173	0.049
Double-outlet right ventricle	0.160	0.166	0.085	0.071	0.066	0.102
CHD (subtypes not distinguished)	0.721	0.663	0.692	0.270	0.652	0.586

External Test Set from Center-B

CHD Subtypes	CHDdECG	CNN	LSTM	*k*-NN	RF	Xgboost
Atrial septal defect	0.264	0.278	0.218	0.091	0.221	0.221
Patent ductus arteriosus	0.114	0.092	0.094	0.041	0.144	0.152
Ventricular septal defect	0.288	0.256	0.197	0.079	0.202	0.266
CHD (subtypes not distinguished)	0.464	0.424	0.413	0.132	0.412	0.461

External Test Set from Center-C

CHD Subtypes	CHDdECG	CNN	LSTM	*k*-NN	RF	Xgboost
Atrial septal defect	0.717	0.665	0.686	0.501	0.696	0.585
Patent ductus arteriosus	0.303	0.265	0.291	0.128	0.252	0.208
Anomalous origin of a coronary artery	0.043	0.056	0.096	0.105	0.065	0.043
Ventricular septal defect	0.531	0.492	0.398	0.286	0.431	0.436
Coarctation of the aorta	0.035	0.085	0.071	0.028	0.021	0.014
Pulmonary atresia	0.119	0.103	0.148	0.103	0.096	0.103
Single ventricle	0.097	0.090	0.062	0.065	0.102	0.082
Tetralogy of fallot	0.388	0.312	0.343	0.193	0.298	0.395
Atrioventricular septal defect	0.060	0.057	0.035	0.047	0.028	0.041
CHD (subtypes not distinguished)	0.815	0.698	0.732	0.480	0.682	0.610

Table S4. Classification performances measured by "PR-AUC" on internal and external test sets.

Internal Test Set from Center-A

Characteristic	Stratification	Count	Prop	ROC-AUC	PR-AUC	Spec	Sens	Brier
Sex	male	7758	64.65%	0.931	0.764	0.907	0.805	0.0837
	female	4242	35.35%	0.918	0.764	0.904	0.791	0.0887
Age (year)	< 1	2876	23.97%	0.936	0.773	0.894	0.824	0.0873
	1-2	2648	22.07%	0.922	0.785	0.917	0.797	0.0826
	2-3	1829	15.24%	0.907	0.719	0.911	0.755	0.0896
	3-4	4647	38.73%	0.931	0.768	0.904	0.805	0.0844
Heart rate (bpm)	< 60	233	1.94%	0.954	0.643	0.915	0.844	0.0731
	60-80	336	2.80%	0.892	0.737	0.855	0.803	0.1248
	81-100	1651	13.76%	0.898	0.699	0.853	0.781	0.1158
	101-120	2982	24.85%	0.921	0.764	0.906	0.793	0.0855
	121-140	3035	25.29%	0.935	0.783	0.916	0.803	0.0800
	141-160	2442	20.35%	0.935	0.798	0.924	0.824	0.0722
	> 160	1321	11.01%	0.934	0.758	0.920	0.781	0.0769

External Test Set from Center-B

Characteristic	Stratification	Count	Prop	ROC-AUC	PR-AUC	Spec	Sens	Brier
Sex	male	3679	51.55%	0.929	0.487	0.933	0.803	0.0576
	female	3458	48.45%	0.902	0.446	0.940	0.732	0.0562
Age (year)	< 1	2171	35.46%	0.949	0.479	0.937	0.819	0.0542
	1-2	1871	24.93%	0.903	0.429	0.932	0.705	0.0607
	2-3	1998	21.27%	0.905	0.525	0.943	0.754	0.0545
	3-4	2081	18.34%	0.897	0.460	0.935	0.794	0.0596
Heart rate (bpm)	< 60	99	0.21%	0.923	0.417	0.923	1.000	0.0808
	60-80	338	1.61%	0.896	0.817	0.921	0.564	0.1294
	81-100	1502	7.97%	0.892	0.491	0.937	0.714	0.0649
	101-120	2022	17.46%	0.898	0.495	0.934	0.746	0.0619
	121-140	1813	27.03%	0.938	0.472	0.938	0.883	0.0551
	141-160	1463	27.84%	0.925	0.434	0.935	0.800	0.0528
	> 160	884	17.88%	0.925	0.480	0.941	0.844	0.0506

External Test Set from Center-C

Characteristic	Stratification	Count	Prop	ROC-AUC	PR-AUC	Spec	Sens	Brier
Sex	male	4398	54.16%	0.903	0.802	0.906	0.776	0.1086
	female	3723	45.84%	0.913	0.832	0.908	0.798	0.0993
Age (year)	< 1	2171	26.73%	0.900	0.812	0.906	0.792	0.1066
	1-2	1871	23.04%	0.907	0.810	0.897	0.786	0.1058
	2-3	1998	24.60%	0.907	0.803	0.903	0.787	0.1051
	3-4	2081	25.62%	0.916	0.833	0.921	0.777	0.0999
Heart rate (bpm)	< 60	99	1.22%	0.843	0.547	0.851	0.800	0.1030
	60-80	338	4.16%	0.885	0.848	0.774	0.885	0.1330
	81-100	1502	18.50%	0.898	0.867	0.876	0.823	0.1204
	101-120	2022	24.90%	0.909	0.812	0.913	0.794	0.0951
	121-140	1813	22.32%	0.898	0.751	0.913	0.742	0.1037
	141-160	1463	18.02%	0.912	0.799	0.921	0.762	0.0995
	> 160	884	10.89%	0.917	0.848	0.944	0.730	0.0966

Table S5. Stratification analyses of individuals and CHDdECG's detection effect verification on an internal and two external test sets. ROC-AUC: receiver operating characteristic – area under the curve; PR-AUC: precision-recall – area under the curve; Spec: specificity; Sens: sensitivity; Brier: brier score.
