# Supplementary material for: Congenital heart disease detection by pediatric electrocardiogram based deep learning integrated with human concepts
Source: Nat Commun. 2024 Feb 1;15:976. doi: 10.1038/s41467-024-44930-y (PMC10834950; doi:10.1038/s41467-024-44930-y)
Supplement: Supplementary file 2 — Reporting Summary [file 41467_2024_44930_MOESM2_ESM.pdf]

nature portfolio

Corresponding author(s): Huiying Liang

Last updated by author(s): Dec 6, 2023

Reporting Summary

Nature Portfolio wishes to improve the reproducibility of the work that we publish. This form provides structure for consistency and transparency in reporting. For further information on Nature Portfolio policies, see our Editorial Policies and the Editorial Policy Checklist.

Statistics

For all statistical analyses, confirm that the following items are present in the figure legend, table legend, main text, or Methods section.

n/a	Confirmed	
☐	☒	The exact sample size (*n*) for each experimental group/condition, given as a discrete number and unit of measurement
☐	☒	A statement on whether measurements were taken from distinct samples or whether the same sample was measured repeatedly
☒	☐	The statistical test(s) used AND whether they are one- or two-sided<br>*Only common tests should be described solely by name; describe more complex techniques in the Methods section.*
☒	☐	A description of all covariates tested
☒	☐	A description of any assumptions or corrections, such as tests of normality and adjustment for multiple comparisons
☐	☒	A full description of the statistical parameters including central tendency (e.g. means) or other basic estimates (e.g. regression coefficient) AND variation (e.g. standard deviation) or associated estimates of uncertainty (e.g. confidence intervals)
☐	☒	For null hypothesis testing, the test statistic (e.g. *F*, *t*, *r*) with confidence intervals, effect sizes, degrees of freedom and *P* value noted<br>*Give P values as exact values whenever suitable.*
☒	☐	For Bayesian analysis, information on the choice of priors and Markov chain Monte Carlo settings
☐	☒	For hierarchical and complex designs, identification of the appropriate level for tests and full reporting of outcomes
☒	☐	Estimates of effect sizes (e.g. Cohen's *d*, Pearson's *r*), indicating how they were calculated

Our web collection on statistics for biologists contains articles on many of the points above.

Software and code

Policy information about availability of computer code

Data collection	In this study, a total of three data sets, including a comprehensive dataset used for model training, validation, and internal testing from Center-A, an external test set from Center-B, and another external test data set from Center-C, were used to development and broadly test the model. Center-A and Center-B are two different referral centers from the same tertiary hospital located in Southern China. Center-C is a large children's medical center located in Northeast China. Furthermore, the ECG data in Center-A and Center-B were collected using identical ECG devices (GE MAC800) from August 2014 to October 2020, while the data in Center-C were collected utilizing a distinct brand of ECG device (NIHON KOHDEN ECG-2550) from January 2020 to June 2023.
Data analysis	Matlab R2018a(9.4.0.813654) was used for ECG data processing and Jupyter notebook(6.0.3) with python(3.8.3) was used for data analysis. Code for the algorithm development is open source with no restrictions and available at https://github.com/shuaih720/CHDdECG

For manuscripts utilizing custom algorithms or software that are central to the research but not yet described in published literature, software must be made available to editors and reviewers. We strongly encourage code deposition in a community repository (e.g. GitHub). See the Nature Portfolio guidelines for submitting code & software for further information.

Data

Policy information about availability of data

All manuscripts must include a data availability statement. This statement should provide the following information, where applicable:

- Accession codes, unique identifiers, or web links for publicly available datasets
- A description of any restrictions on data availability
- For clinical datasets or third party data, please ensure that the statement adheres to our policy

All data supporting the findings described in this manuscript are available in the article and in the Supplementary Information or/and from the corresponding author upon request. The ECG data used in this study cannot be shared publicly due to privacy restrictions. However, in the case of non-commercial use, researchers can sign the Data Access Form and Data License provided at the Github repository (https://github.com/shuaih720/CHDdECG) and contact H. Liang (lianghuiying@hotmail.com) to access the de-identified representative ECG data. Generally, we will respond within one week. Access will be granted by the data access committee. We have also deposited some representative data at the Github repository (https://github.com/shuaih720/CHDdECG), which is publicly available for scientific research and non-commercial use. Source data used to generate the tables and figures are provided with this paper.

Human research participants

Policy information about studies involving human research participants and Sex and Gender in Research.

Reporting on sex and gender	Training&Validation dataset from Center-A: 65869 cases, 23873 female(4126 cases diagnosed with congenital heart disease) and 41996 male(6801 cases diagnosed with congenital heart disease)<br>Test dataset from Center-A: 12000 cases, 4242 female(757 cases diagnosed with congenital heart disease) and 7758 male (1281 cases diagnosed with congenital heart disease)<br>External test dataset from Center-B: 7,137 cases, 3458 female(138 cases diagnosed with congenital heart disease) and 3679 male(162 cases diagnosed with congenital heart disease)<br>External test dataset from Center-C: 8,121 cases, 3,723 females(1126 cases diagnosed with congenital heart disease) and 4,398 males(1395 cases diagnosed with congenital heart disease)
Population characteristics	The electrocardiogram cases were independently collected in an unbiased manner from the electronic medical record system at three referral centers of two large tertiary care hospitals in China. And the detailed population characteristics are listed in the Table 2 in the supplementary material.
Recruitment	Samples were chosen as unbiased representations of participants served at each of the three centers in this research, without regards to demographic or clinical covariates.
Ethics oversight	This study was approved by the Medical Ethics Committee of Guangdong Provincial People's Hospital (KY-Q-2022-144-01). In accordance with ethical guidelines, this study secured a waiver for informed consent based on its retrospective analysis of anonymized data, ensuring privacy and security without explicit consent from subjects.

Note that full information on the approval of the study protocol must also be provided in the manuscript.

Field-specific reporting

Please select the one below that is the best fit for your research. If you are not sure, read the appropriate sections before making your selection.

☐ Life sciences ☒ Behavioural & social sciences ☐ Ecological, evolutionary & environmental sciences

For a reference copy of the document with all sections, see nature.com/documents/nr-reporting-summary-flat.pdf

Behavioural & social sciences study design

All studies must disclose on these points even when the disclosure is negative.

Study description	We developed and validated the performance of a Deep Neural Network for the detection of congenital heart diseases based on electrocardiogram data. The data in the study is quantitative consisting of electrocardiogram records and their corresponding annotation.
Research sample	The training &validation dataset from Center-A was a deidentified, retrospective dataset of young children with an average age of 2.12±1.50 years. Additionally, the test dataset from Center-A had an average age of 2.13±1.23 years, the external test dataset from Center-B had an average age of 1.55±1.04 years, and the external test dataset from Center-C had an average age of 1.95±1.17 years. This is the age distribution of all the data we can get from multiple centers involved in this study, and intervening in congenital heart disease at this stage can also yield relatively good results.
Sampling strategy	All ECG data available to us, after undergoing exclusion criteria, have been utilized in this study. Common congenital heart diseases, such as atrial septal defect and ventricular septal defect, have substantial sample sizes ranging from 700 to 4500 cases. Even some

	rare congenital heart diseases, such as Double-outlet right ventricle and Anomalous origin of a coronary artery, are represented by around one hundred cases. The volume of data and coverage of subtypes of congenital heart diseases surpass those in previous related studies.
Data collection	In this study, a total of three data sets, including a comprehensive dataset used for model training, validation, and internal testing from Center-A, an external test set from Center-B, and another external test data set from Center-C, were used to development and broadly test the model. Center-A and Center-B are two different referral centers from the same tertiary hospital located in Southern China. Center-C is a large children's medical center located in Northeast China. Furthermore, the ECG data in Center-A and Center-B were collected using identical ECG devices (GE MAC800) from August 2014 to October 2020, while the data in Center-C were collected utilizing a distinct brand of ECG device (NIHON KOHDEN ECG-2550) from January 2020 to June 2023. In the experiment comparing the cardiologist and the CHDdECG model, the cardiologist was blinded to the CHDdECG's diagnosis, but could compare the human expert's diagnosis with the CHDdECG's diagnosis after the diagnosis. Blinding in the remaining experiments was not relevant to our study.
Timing	The ECG data in Center-A and Center-B were collected from August 2014 to October 2020, while the data in Center-C were collected from January 2020 to June 2023
Data exclusions	(i) 87,322 cases in Center-A, 18,331 cases in Center-B, and 9,982 cases in Center-C with diagnostic label missing were excluded;<br>(ii) 47,854 cases in Center-A, 10,332 cases in Center-B, and 4,632 cases in Center-C with corrupted ECG data were excluded;<br>(iii) 110,202 cases in Center-A, 21,095 cases in Center-B, and 11,361 cases in Center-C obtained after intervention were excluded;<br>(iv) 18,198 ECG data in Center-A, 2,890 ECG data in Center-B, and 4,765 ECG data in Center-C from the resampled individuals were excluded.
Non-participation	We did not require informed consent for this study given that the data was fully deidentified.
Randomization	Our data was selected from the study period according to the selection strategy mentioned in the "Data Collection" section. And The cases were randomly assigned to training&validation dataset,test dataset, and external test dataset.

Reporting for specific materials, systems and methods

We require information from authors about some types of materials, experimental systems and methods used in many studies. Here, indicate whether each material, system or method listed is relevant to your study. If you are not sure if a list item applies to your research, read the appropriate section before selecting a response.

Materials & experimental systems

n/a	Involved in the study
☒	☐ Antibodies
☒	☐ Eukaryotic cell lines
☒	☐ Palaeontology and archaeology
☒	☐ Animals and other organisms
☒	☐ Clinical data
☒	☐ Dual use research of concern

Methods

n/a	Involved in the study
☒	☐ ChIP-seq
☒	☐ Flow cytometry
☒	☐ MRI-based neuroimaging
